# SOFA-2 reclassification: data availability and incremental prognostic value

**DOI:** 10.1186/s13054-026-06279-0

**Published:** 2026-08-20

**Authors:** Dilek Kuzukıran Kocataş

**Affiliations:** Department of Anesthesiology and Reanimation, Division of Intensive Care Medicine, Ankara Training and Research Hospital, Ankara, Türkiye

**To the Editor,**

We read with interest the study by Ono et al. examining admission-level reclassification between the original Sequential Organ Failure Assessment (SOFA) score and SOFA-2 and the association between score discordance and intensive care unit (ICU) mortality in the large, multicenter OneICU cohort [1]. The presentation of subscore transitions using Sankey diagrams and heat maps provides valuable insight into how the transition to SOFA-2 may change the classification of organ dysfunction. However, three issues warrant clarification when interpreting the incremental prognostic value of these findings: differences in component data availability between the two scores, the specification of inverse probability weighting, and the prognostic interpretation of the exact-matching analyses.

First, the issue is not merely that some component data were missing, but that data availability differed substantially between the two scores. Supplementary Table 1 reports that all variables required to calculate the respiratory component were unavailable in 40,576 admissions (30.2%) for the original SOFA score, compared with only 354 admissions (0.26%) for SOFA-2. For the neurologic component, the corresponding proportions were 12.9% and 2.8%. According to the Supplementary Methods, an unmeasured component was assigned a score of zero until a measurement became available [1].

The ability of SOFA-2 to use the peripheral oxygen saturation to inspired oxygen fraction ratio (SpO₂/FiO₂) when the arterial oxygen partial pressure to inspired oxygen fraction ratio (PaO₂/FiO₂) is unavailable and the motor component of the Glasgow Coma Scale under appropriate conditions is a deliberate advantage intended to make better use of routinely available clinical data. Nevertheless, this feature means that the observed reclassification may reflect not only differences in score definitions but also differences in the data required by the two scores. Assigning zero when the measurements required for an original SOFA component are unavailable may result in lower recorded original SOFA scores and may partly contribute to reclassification toward a positive dSOFA, defined as SOFA-2 minus SOFA. Because measurement practices may vary with illness severity and the need for organ support, the observed differences may also reflect informative measurement patterns. The authors acknowledge unmeasured components as a limitation, but the key concern is that differences in data availability may not affect the two scores equally.

A complete-case analysis would itself be susceptible to selection bias. It may therefore be more informative to present respiratory and neurologic subscore transitions separately for admissions with and without the measurements required for the original SOFA score. Such a sensitivity analysis could help distinguish the relative contributions of differences in score definitions and asymmetric data availability to the observed reclassification. A related operational difference concerns the renal component: the SOFA-2 criterion regarding renal replacement therapy for “non-renal use” was not applied [1]. Reporting the number of admissions affected by this modification, together with a sensitivity analysis excluding patients receiving renal replacement therapy, could clarify its influence on renal reclassification.

Second, the variables used for inverse probability weighting across dSOFA categories and the target estimand of the weighted comparison are not fully specified. Supplementary Fig. 9 presents balance before and after weighting for demographic characteristics and measures of illness severity, as well as vasopressors, respiratory support, mechanical circulatory support, and renal replacement therapy. However, it is unclear whether the variables assessed in this balance plot were the same variables included in the multinomial propensity-score model [1].

This distinction is important because some of these organ-support and vasoactive variables contribute directly to the definition of SOFA or SOFA-2 subscores and, consequently, to dSOFA classification. If these variables were included in the model, their timing and role should be specified, because conditioning on variables that contribute to dSOFA classification could complicate interpretation of the target estimand. If they were not included, the set of variables underlying the balance assessment in Supplementary Fig. 9 should be clarified.

Furthermore, dSOFA is not a treatment or intervention but the difference between two deterministic scores. The purpose of the weighted comparison and the interpretation assigned to it should therefore be stated explicitly. When propensity-score weighting is used, reporting the variables included in the model, the target estimand, the distribution and handling of extreme weights, and the effective sample size is important for interpreting the analysis [2]. Even if weighting balances measured characteristics, it cannot by itself resolve classification problems arising from differential data availability.

Third, the reciprocal exact-matching analyses demonstrate an association between score discordance and mortality but do not directly establish that using both scores provides incremental prognostic value. Because dSOFA is defined as SOFA-2 minus SOFA, variation in dSOFA at a fixed SOFA-2 value necessarily represents variation in the opposite direction in the original SOFA score. In the groups matched on SOFA-2, the mean SOFA-2 score was 7.41 in all three groups, whereas the mean original SOFA scores were 10.2, 7.4, and 4.7 in the negative, minimal, and positive dSOFA groups, respectively. The same algebraic relationship applies to SOFA-2 when matching is performed on the original SOFA score [1].

These findings show that admissions assigned the same level of severity by one score may remain heterogeneous with respect to the other score and mortality. The matching analyses are informative in this regard. However, incremental prognostic value requires direct assessment of whether information from score discordance improves prediction beyond either score alone. This could be examined by comparing prespecified models based on SOFA and SOFA-2 and evaluating whether information from score discordance improves discrimination, calibration, and overall prediction error under appropriate validation [3]. If “complementarity” instead refers to a more comprehensive clinical description of organ dysfunction, score discordance should be evaluated against an independent clinical reference or in terms of its effect on clinical decision-making. The current matching analyses do not distinguish between prognostic and clinical complementarity.

The study convincingly demonstrates that SOFA-2 produces meaningful changes in the classification of organ dysfunction. Further analyses addressing differential data availability, clarifying the weighting strategy, and directly evaluating incremental prognostic performance would help determine whether considering discordance between SOFA and SOFA-2 provides additional prognostic or clinical value.

## Data Availability

No datasets were generated or analysed during the current study.

## Declarations

### Authors’ information

Dilek Kuzukıran Kocataş is an intensive care medicine physician at Ankara Training and Research Hospital, Ankara, Türkiye.

## Abbreviations

dSOFA: Difference between SOFA-2 and the original SOFA score
FiO₂: Fraction of inspired oxygen
ICU: Intensive care unit
PaO₂: Partial pressure of arterial oxygen
SOFA: Sequential Organ Failure Assessment
SpO₂: Peripheral oxygen saturation
